# Association of Grain Iron and Zinc Content With Other Nutrients in Pearl Millet Germplasm, Breeding Lines, and Hybrids

**DOI:** 10.3389/fnut.2021.746625

**Published:** 2022-02-02

**Authors:** Mahalingam Govindaraj, Anand Kanatti, Kedar Nath Rai, Wolfgang H. Pfeiffer, Harshad Shivade

**Affiliations:** ^1^International Crops Research Institute for the Semi-arid Tropics (ICRISAT), Patancheru, India; ^2^Alliance of Bioversity International and the International Center for Tropical Agriculture (CIAT), Cali, Colombia; ^3^HarvestPlus Program, International Food Policy Research Institute (IFPRI), Washington, DC, United States

**Keywords:** biofortification, iron, macronutrients, micronutrients, pearl millet, zinc

## Abstract

Micronutrient deficiency is most prevalent in developing regions of the world, including Africa and Southeast Asia where pearl millet (*Pennisetum glaucum* L.) is a major crop. Increasing essential minerals in pearl millet through biofortification could reduce malnutrition caused by deficiency. This study evaluated the extent of variability of micronutrients (Fe, Zn, Mn, and Na) and macronutrients (P, K, Ca, and Mg) and their relationship with Fe and Zn content in 14 trials involving pearl millet hybrids, inbreds, and germplasm. Significant genetic variability of macronutrients and micronutrients was found within and across the trials (Ca: 4.2–40.0 mg 100 g^−1^, Fe: 24–145 mg kg^−1^, Zn: 22–96 mg kg^−1^, and Na: 3.0–63 mg kg^−1^). Parental lines showed significantly larger variation for nutrients than hybrids, indicating their potential for use in hybrid parent improvement through recurrent selection. Fe and Zn contents were positively correlated and highly significant (*r* = 0.58–0.81; *p* < 0.01). Fe and Zn were positively and significantly correlated with Ca (*r* = 0.26–0.61; *p* < 0.05) and Mn (*r* = 0.24–0.50; *p* < 0.05). The findings indicate that joint selection for Fe, Zn, and Ca will be effective. Substantial genetic variation and high heritability (>0.60) for multiple grain minerals provide good selection accuracy prospects for genetic enhancement. A highly positive significant correlation between Fe and Zn and the nonsignificant correlation of grain macronutrients and micronutrients with Fe and Zn suggest that there is scope to achieve higher levels of Fe/Zn simultaneously in current pearl millet biofortification efforts without affecting other grain nutrients. Results suggest major prospects for improving multiple nutrients in pearl millet.

## Introduction

Micronutrient malnutrition affects more than two billion people worldwide (1–4). The most prevalent forms of malnutrition are those arising from deficiencies of iron (Fe), zinc (Zn), vitamin A, and iodine (I), which occur particularly among women and children in developing countries. In these countries, more than 40% of preschool children are stunted because of Zn deficiency, whereas 30% of preschool children are anemic because of Fe deficiency (5, 6). For instance, India loses about 4 million children every year to disability-adjusted life years (DALYs) caused by Fe deficiency or anemia (7), with another 2.8 million children lost to DALYs because of stunted growth caused by Zn deficiency (8, 9). Humans require more than 40 nutrients that are essential to meet the metabolic needs of the body including proteins, lipids, macronutrients, micronutrients, and vitamins. Inadequate consumption of any of these will result in adverse metabolic disturbances, leading to sickness, poor health, impaired development in children, and a large economic cost to society (1). Men and women aged between 25 and 50 years require a daily intake of 800 mg of calcium (Ca) and phosphorus (P), 280–350 mg of magnesium (Mg), 2,000 mg of potassium (K), 10–15 mg of Fe and Zn, 2–5 μg of manganese (Mn), and 500 mg of sodium (Na) to meet the Recommended Dietary Allowance (RDA) (10–12). This is reason enough for developing public health policies that encourage the consumption of micronutrients at the RDA levels. Evidence suggests that the main cause of hidden hunger in developing countries is the unavailability of essential minerals in staple diets, particularly those comprising cereal-based foods that are inherently low in micronutrients such as Fe, Zn, and vitamin A (13–16). Efforts are underway to breed for increased Fe, Zn, and vitamin A content (17, 18). The agronomic or genetic enhancement of essential micronutrients and vitamins in edible parts of staple food crops is called biofortification. Genetic biofortification is a one-time investment and has no genetic erosion such as the dwarfing genes that catalyzed the Green Revolution in wheat and rice. Biofortification breeding is currently limited to a few crops, including iron-fortified pearl millet and beans, zinc-fortified wheat, rice, and maize, and vitamin A-fortified orange sweet potato, cassava, and maize (18) (www.harvestplus.org). There are biofortification initiatives in other crops such as lentils (19).

Pearl millet is grown on 26 million ha globally, of which 7.4 m ha are in the most marginal arid and semiarid tropical regions of India, particularly in Maharashtra, Rajasthan, Gujarat, and Uttar Pradesh states (20). It is an important staple food for millions of people and a major source of dietary energy and nutritional security for the vast rural communities in these regions (21). Pearl millet is also the cheapest source of not only energy and protein but also of Fe and Zn (22). Given its high nutritional value, pearl millet can contribute significantly to improve the nutritional status of millions. However, all the released and commercially grown pearl millet cultivars have low levels of micronutrients, especially low Fe (42 mg kg^−1^) and Zn (32 mg kg^−1^) (23). A few studies (24, 25) have reported crop breeding efforts that have significantly contributed to improving grain yield in commercial cultivars, but with reduced grain nutrient concentrations compared to landraces. The International Crops Research Institute for the Semi-Arid Tropics (ICRISAT) initiated biofortification research under the umbrella of the HarvestPlus Challenge Program of the CGIAR, to develop high-Fe open-pollinated varieties (OPVs), improved breeding lines, and hybrid parents for high Fe and Zn contents. Wide variability for Fe and Zn contents and their genetic inheritance are well documented (26, 27). These two micronutrients are governed by additive-effect genes (23, 26, 28). Among the micronutrients, Fe and Zn can be significantly improved through biofortification breeding. While the pattern of association between Fe and Zn is being studied, the association of these two traits with other important macro- and micronutrients has not been studied extensively in sizeable pearl millet breeding materials. As part of the HarvestPlus-supported biofortification program, this study assessed the available variability for grain micronutrients (Mn, Na) and macronutrients (P, K, Ca, and Mg) and their relationship with Fe and Zn content (current biofortification target nutrients) in different pearl millet breeding trials, including germplasm accessions, hybrid parents, and commercial cultivars to develop cultivars with improved iron and zinc content.

## Materials and Methods

### Field Trial

This study consisted of 928 entries in 14 replicated trials during the 2012–2013 crop season in India. The details of the experimental materials and trials are given in Table 1. All these field trials were evaluated during the rainy season using a randomized complete block design with two replications (trials 7 and 8 were replicated thrice) in an Alfisol precision field at ICRISAT, Hyderabad, India (latitude: 17.51° N, longitude: 78.27° E, altitude: 545 m) (Table 1). Entries in trials 1–4 were planted in two rows of 4 m-long plots. Entries in the remaining trials were planted in one-row 2 m-long plots, with an interrow spacing of 75 cm and intrarow spacing of 15 cm. In all the field trials, fertilizer was applied as per standard recommendations for the site to maintain good soil fertility of the experimental fields to ensure trial precision. Open-pollinated main panicles of five random plants with good seed sets were harvested from each plot at or after physiological maturity in all the trials. The harvested panicles were sun-dried on a tarpaulin sheet for 12–15 days, stored in cloth bags, hand threshed, and the grains were divested of glumes and foreign matter, if any, to produce grain samples for laboratory analyses.

**Table 1:** Study materials evaluated in 14 trials and remarks.

**Trial No**.	**Name of the trial**	**No. of entries**	**No. of replications**	**Remarks**
Trial-1	Commercial hybrid trial	40	2	Released and commercially grown hybrids
Trial-2	Hybrid trial-1	39	2	Pipeline hybrids
Trial-3	Hybrid trial-2	36	2	Pipeline hybrids
Trial-4	Hybrid trial-3	28	2	Pipeline hybrids
Trial-5	Hybrid trial-4	30	2	Pipeline hybrids
Trial-6	Released cultivar trial	130	2	Released cultivars at the national level since 1970s
Trial-7	Hybrid parental trial-1	45	3	Inbred/hybrid parents
Trial-8	Hybrid parental trial-2	40	3	Inbred/hybrid parents
Trial-9	Testcross parental trial-1	72	2	Inbred/hybrid parents
Trial-10	Testcross parental trial-2	76	2	Inbred/hybrid parents
Trial-11	Testcross parental trial-3	66	2	Inbred/hybrid parents
Trial-12	Testcross parental trial-4	56	2	Inbred/hybrid parents
Trial-13	*Iniadi* accessions	200	2	Germplasm accessions
Trial-14	Designated B-lines	70	2	Mainstream seed parent

### Mineral Estimation

Grain macronutrients such as P, K, Ca, and Mg and micronutrients such as Fe, Zn, Mn, and Na were analyzed following the methods described by Wheal et al. (29) at Waite Analytical Laboratory, Adelaide University, Australia. Grain samples were finely ground and oven-dried at 60°C for 48 h before analyzing their nutrient content. This help to reduce the uniform moisture of the grain samples at ~12%. The ground samples (0.2 g) were transferred to 25 ml polypropylene Plasma Preparation Tube (PPT) tubes and digestion was initiated by adding 2.0 ml of concentrated nitric acid (HNO_3_) and 0.5 ml of 30% hydrogen peroxide (H_2_O_2_). Tubes were vortexed to ensure the entire sample was wetted and then predigested overnight at room temperature. Tubes were vortexed again before being placed in the digestion block. They were initially heated at 80°C for 1 h followed by digestion at 120°C for 2 h. After digestion, the volume of the digest was brought to 25 ml using distilled water and the content was agitated for a minute in the vortex mixer. The digests were filtered and the nutrient content was determined using Inductively Coupled Plasma Optical Emission Spectroscopy (ICP-OES). Estimation of aluminum (Al) as an index of soil or dust contamination was done in the grain samples of all the trials using the procedure followed in wheat (30).

### Data Analyses

Data analysis was done using SAS University Edition (SAS/STAT®, SAS Institute Incorporation, Cary, North Carolina, USA). The analyses of variance of all the trials were done following Gomez and Gomez (31). This study applied the Generalized linear model (GLM) statistical analysis since most of the trials consisted of fixed-line materials (no early stages of a selection). Broad-sense heritability (H^2^) was calculated following Hallauer et al. (32). Correlation analysis among grain minerals in all the trials was done as per Al-Jibouri et al. (33) and the significance of the correlation coefficients was tested using the standard table in Snedecor and Cochran (34). Genotype (G) and traits (T) analysis were performed using the “Genotype-by-Trait” module of the genotypes, and genotype × environment interaction (GGE) biplot software (35) (http://ggebiplot.com/biplot-breeder's_kit.htm).

## Results and Discussion

This study emphasized total variability for multiple grain nutrients to establish a baseline for most nutrients in pearl millet. Therefore, the results and their interpretation mostly focused on the magnitude of variability of each trial (hybrid parents, commercial/released hybrids, and germplasm accession) and Fe/Zn association with other nutrients under highly managed precision fields. All the 14 trials had quality data, as indicated by the Coefficient of variation (CV)% of each trial. The magnitude and significance of genetic variability are prerequisites for an effective pre-breeding program enabled through the efficient selection of these minerals for genetic improvement.

### Genetic Variability and Heritability for Grain Mineral Contents

**Figure 1:**
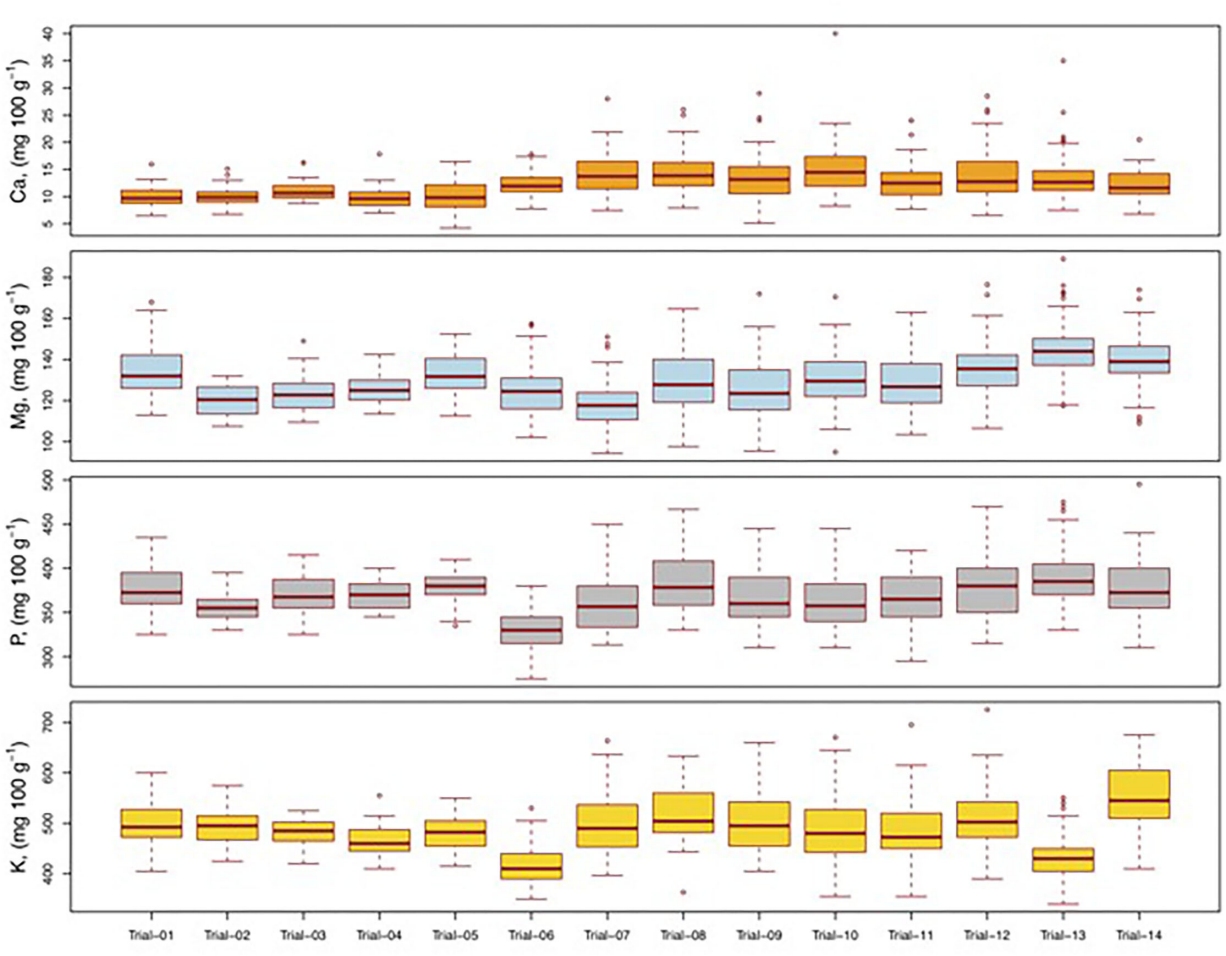
Variability for potassium (K), phosphorus (P), magnesium (Mg), and calcium (Ca) in pearl hybrid trials (trial 1 to trial 6), inbred trials (trial 7 to trial 12 and trial 14), and germplasm accessions (trial 13) at the ICRISAT, Hyderabad, India.

**Figure 2:**
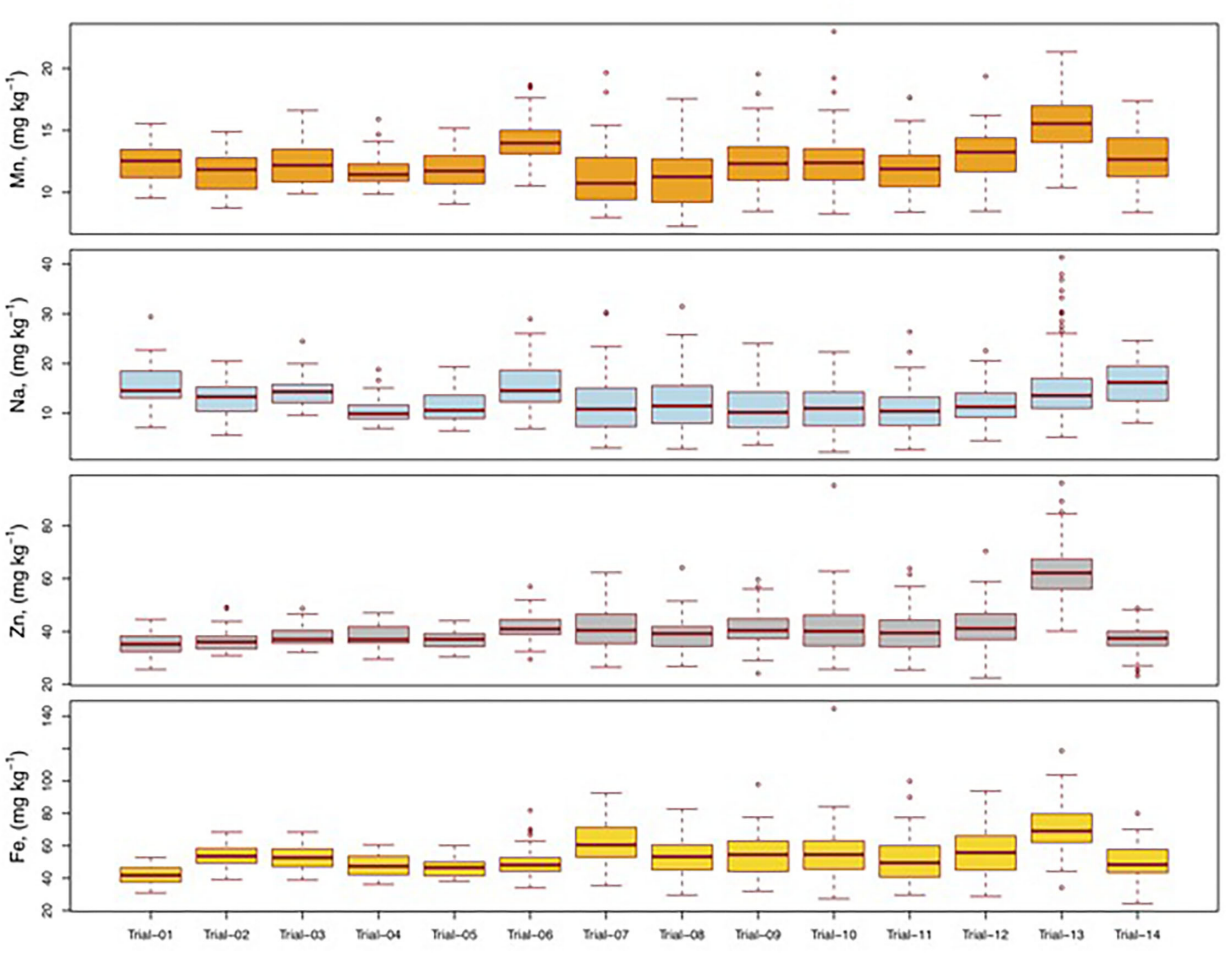
Variability for iron (Fe), zinc (Zn), sodium (Na), and manganese (Mn) in pearl millet hybrid trials (trial 1 to trial 6), inbred trials (trial 7 to trial 12 and trial 14), and germplasm accessions (trial 13) at the ICRISAT, Hyderabad, India.

The analysis of variance showed that the differences among the genotypes were highly significant for Fe and Zn in all the trials. Variation attributable to genotypes was not significant for Mn, Ca, Na, and Mg in one trial and P and K in two trials. Significant genotypic differences were also observed for other grain mineral content (Table 2). The nonsignificant values observed for very few macronutrients in three trials were not expected. This could possibly be because the trial consisted of genotypes that had been selected either for grain yield traits or partially for grain micronutrient (Fe/Zn) content during line development. A further investigation of these specific pedigrees and genetic backgrounds is warranted for a better understanding of variability. The results also showed that compared to other minerals, there was substantial genetic variability for Fe and Zn in the elite materials. For instance, the genotypes in the commercial hybrid trial, released cultivar trial, and designated parents of the seed (designated B-lines) trial were directly selected for grain yield and its components, whereas those in the other trials were mainly selected for Fe and Zn contents. Across the 14 trials, the means of P, K, Ca, and Mg content were 369, 489, 12, and 130 mg 100 g^−1^, respectively (Figure 1; Supplementary Table 1). The variability for these macronutrients across the 14 trials ranged from 275 to 495 mg 100 g^−1^ for P, 340–725 mg 100 g^−1^ for K, 4–40 mg 100 g^−1^ for Ca, and 94–189 mg 100 g^−1^ for Mg. Similarly, the mean micronutrient content across the 14 trials was 53 mg kg^−1^ for Fe, 41 mg kg^−1^ for Zn, and 13 mg kg^−1^ for both Mn and Na (Figure 2; Supplementary Table 2). The magnitude of variability was higher for macronutrients than for micronutrients. Mean and variability range of eight minerals were in the order K > P > Mg > Ca > Fe > Zn > Na > Mn. The results also revealed larger variability for P, K, Ca, Mg, Fe, Zn, Mn, and Na in parents/inbred trials compared to the hybrid trials. The variation for macronutrients (P, K, Ca, and Mg) in parent/inbred trials ranged from 25 to 157% and the variation for micronutrients (Fe, Zn, Mn, and Na) in parent/inbred trials ranged from 16 to 139% compared to those observed in the hybrid trials. These significant differences in grain mineral content among diverse sets of genetic materials suggested the promising prospect of enhancing these mineral nutrients in pearl millet, in addition to Fe and Zn. Previous studies in pearl millet have revealed wide genetic variability in grain micronutrient contents of Fe and Zn to be highly heritable (26, 36–39). A couple of studies have reported breeding approaches that have significantly improved grain yield in commercial cultivars, but reduced grain nutrient concentrations compared to old cultivars (24, 25). This study revealed the presence of adequate variation for Fe and Zn in elite genetic backgrounds for further breeding. All these lines were initially bred for yield-related traits as a part of mainstream breeding and subsequently screened for micronutrients. This showed the prospects for genetic enhancement of pearl millet with respect to these grain minerals, along with productivity traits, which would further make pearl millet a cheap source of Fe/Zn.

**Table 2:** Analysis of variance for macronutrient and micronutrient contents in pearl millet breeding trials at ICRISAT, Hyderabad, India.

**Trial no**.	**Trial name**		**P**	**K**	**Ca**	**Mg**	**Fe**	**Zn**	**Mn**	**Na**
**Hybrid trials**										
Trial-1	Commercial hybrid trial	F-test	^**^	^**^	^**^	^**^	^*^	^**^	^**^	NS
		CV %	4.0	3.7	14.0	3.9	11.6	7.9	7.8	13.8
Trial-2	Hybrid trial-1	F-test	^*^	^**^	^**^	^**^	^*^	^**^	^**^	^**^
		CV %	4.2	4.8	13.6	6.0	10.8	7.6	9.0	13.3
Trial-3	Hybrid trial-2	F-test	^*^	^*^	^**^	^**^	^**^	^**^	^**^	^**^
		CV %	5.5	10.7	12.5	6.1	11.8	7.6	10.6	17.0
Trial-4	Hybrid trial-3	F-test	^**^	^**^	^**^	^**^	^**^	^**^	^**^	^**^
		CV %	3.3	4.2	11.4	3.1	7.1	7.2	5.8	9.8
Trial-5	Hybrid trial-4	F-test	^**^	^**^	^**^	^**^	^**^	^**^	^**^	^**^
		CV %	4.7	4.2	11.9	2.8	6.8	7.4	6.1	9.6
Trial-6	Released cultivar trial	F-test	^**^	^**^	^**^	^**^	^**^	^**^	^**^	^*^
		CV %	5.5	5.5	10.5	4.5	7.9	5.9	7.7	20.0
**Breeding/parental lines**										
Trial-7	Hybrid parental trial-1	F-test	^**^	^**^	^**^	^**^	^**^	^**^	^**^	^**^
		CV %	5.9	6.0	13.8	6.1	9.7	9.2	10.4	15.6
Trial-8	Hybrid parental trial-2	F-test	^**^	NS	^**^	NS	^**^	^**^	^**^	^**^
		CV %	4.7	5.3	14.6	5.3	10.9	10.1	10.7	17.1
Trial-9	Testcross parental trial-1	F-test	^**^	^**^	^**^	^**^	^**^	^**^	^**^	^**^
		CV %	4.7	4.0	15.1	4.9	10.0	8.7	7.1	18.7
Trial-10	Testcross parental trial-2	F-test	NS	^**^	NS	^*^	^*^	^*^	NS	^**^
		CV %	5.0	5.2	15.4	4.1	11.9	10.6	9.3	35.0
Trial-11	Testcross parental trial-3	F-test	^**^	^**^	^**^	^**^	^**^	^**^	^**^	^**^
		CV %	5.7	5.4	14.3	5.6	9.6	10.1	9.5	16.5
Trial-12	Testcross parental trial-4	F-test	^**^	^**^	^**^	^**^	^**^	^**^	^**^	^**^
		CV %	5.6	6.5	14.8	4.0	10.1	9.9	8.8	14.6
Trial-13	*Iniadi* Accessions	F-test	NS	NS	^**^	^**^	^**^	^**^	^**^	^*^
		CV %	6.3	7.9	18.5	6.1	16.7	12.8	10.9	22.0
Trial-14	Designated B-lines	F-test	^**^	^**^	^**^	^**^	^**^	^**^	^**^	^**^
		CV %	5.7	6.0	11.6	5.4	9.5	9.8	7.9	16.5

*,** *Significant at P < 0.05 and P < 0.01 probability, respectively. NS, Non-significant*.

Heritability estimates provide information about the proportion of phenotypic variation that is genetic and allow for the prediction of genetic gains following selection. In this study, broad-sense heritability (H^2^) estimates, averaged across 14 trials, ranged from 0.58 to 0.73 for macronutrients and from 0.67 to 0.70 for micronutrients (Table 3). This implied that, in general, slightly greater selection progress would be possible for grain micronutrients than for grain macronutrients in pearl millet. The heritability observed in inbred/parental trials was higher than that in hybrid trials for both macronutrients and micronutrients. These high heritability values suggest high genetic gains in phenotypic selection since these micronutrients are largely controlled by additive gene action (26, 28). These results are consistent with earlier studies on progeny phenotypic selection, which was highly effective in improving Fe and Zn in pearl millet (26, 40). Previous studies have reported high H^2^ for grain Fe and Zn contents in pearl millet (38, 41, 42). Therefore, the availability of highly heritable variation for these macronutrients and micronutrients suggests that genetic improvement in pearl millet is highly feasible through progeny selection.

**Table 3:** Heritability estimates (broad sense) for grain macronutrient and micronutrient contents in pearl millet breeding trials at ICRISAT, Hyderabad, India.

**Trial Name**	**Macronutrients**				**Micronutrients**			
	**P**	**K**	**Ca**	**Mg**	**Fe**	**Zn**	**Mn**	**Na**
**Hybrid trials**								
Commercial hybrid trial	0.70	0.82	0.54	0.84	0.42	0.69	0.64	–
Hybrid trial-1	0.37	0.59	0.55	0.39	0.45	0.65	0.67	0.77
Hybrid trial-2	0.37	0.51	0.54	0.47	0.44	0.56	0.55	0.51
Hybrid trial-3	0.57	0.66	0.77	0.73	0.80	0.64	0.79	0.87
Hybrid trial-4	0.43	0.67	0.84	0.88	0.79	0.54	0.81	0.88
Released cultivar trial	0.48	0.63	0.66	0.78	0.77	0.74	0.62	0.74
**Breeding/parental lines**								
Hybrid parental trial-1	0.69	0.82	0.79	0.77	0.82	0.83	0.81	0.92
Hybrid parental trial-2	0.72	–	0.77	–	0.82	0.75	0.77	0.87
Testcross parental trial-1	0.74	0.88	0.78	0.82	0.81	0.78	0.84	0.92
Testcross parental trial-2	–	0.86	–	0.85	0.83	0.82	–	0.90
Testcross parental trial-3	0.57	0.85	0.77	0.75	0.88	0.78	0.73	0.86
Testcross parental trial-4	0.66	0.75	0.82	0.86	0.86	0.80	0.75	0.83
*Iniadi* accessions	–	–	0.57	0.54	0.66	0.46	0.51	0.85
Designated B-lines	0.67	0.74	0.72	0.70	0.84	0.69	0.80	0.67
Across trials	0.58	0.72	0.71	0.73	0.73	0.70	0.72	0.67

*Heritability is not estimated where non-significant variances found for few traits*.

### Association of Iron and Zinc With Other Mineral Content

Correlation among different traits is very important to ensure success in indirect selection in a crop breeding program. Therefore, associations between Fe and Zn and their relationship with other grain macronutrients and micronutrients are critical for the success of the genetic biofortification of pearl millet with respect to Fe and Zn. The correlation coefficient between Fe and Zn ranged from 0.58 to 0.79 in hybrids and from 0.64 to 0.81 in inbred and iniadi germplasm (early maturing, large-seeded, originated from Togo regions of West Africa), with an overall correlation coefficient of 0.79 (Table 4). Three hybrid trials, five inbred trials, and a germplasm trial showed a high magnitude of correlation (*r* ≥ 0.70; *p* < 0.01). This implied that Fe and Zn content were closely linked within the common genomic region or via interconnected physiological mechanisms for their uptake and translocation into grains. A similar positive and significant correlation between Fe and Zn has been reported in earlier studies in pearl millet (28, 43–46), which can be attributed to the co-segregation of alleles for these micronutrients and the co-localization of quantitative trait loci (19). The results of this study suggested breeding for Zn concentration to be the secondary target while maintaining the focus on Fe content in pearl millet.

**Table 4:** Correlation coefficient (r) of grain Fe and Zn density with other nutrients' content in pearl millet breeding trials at ICRISAT, Hyderabad, India.

**Trial Name**	**Grain Fe vs. other nutrients**							**Grain Zn vs. other nutrients**					
	**Zn**	**P**	**K**	**Ca**	**Mg**	**Mn**	**Na**	**P**	**K**	**Ca**	**Mg**	**Mn**	**Na**
**Hybrid trials**													
Commercial hybrid trial	0.70^**^	0.13	−0.08	0.18	−0.12	0.17	0.11	0.10	−0.02	0.16	−0.02	0.02	0.05
Hybrid trial-1	0.58^**^	−0.11	−0.16	0.27	0.19	0.46^**^	−0.35^*^	0.18	−0.33^*^	0.49^**^	0.35^*^	0.41^**^	−0.51^**^
Hybrid trial-2	0.59^**^	0.12	−0.40^*^	0.12	0.16	0.49^**^	−0.30	0.27	−0.22	0.24	0.22	0.32	−0.30
Hybrid trial-3	0.78^**^	−0.01	−0.13	0.00	−0.21	0.38^*^	−0.06	0.01	−0.10	−0.01	−0.23	0.13	−0.27
Hybrid trial-4	0.69^**^	0.00	−0.18	0.49^**^	0.00	0.37^*^	−0.19	0.10	−0.05	0.22	−0.07	0.15	−0.38^*^
Released cultivar trial	0.79^**^	0.40^**^	−0.04	0.30^**^	0.00	0.15	−0.13	0.47^**^	−0.02	0.21^*^	0.12	0.20^*^	−0.02
**Breeding/parental lines**													
Hybrid parental trial-1	0.74^**^	0.26	−0.26	0.21	0.42^**^	0.23	−0.44^**^	0.36^*^	−0.10	0.26	0.52^**^	0.36^*^	−0.37^*^
Hybrid parental trial-2	0.66^**^	−0.01	0.23	−0.17	−0.16	0.41^**^	−0.06	0.19	0.48^**^	−0.11	0.03	0.52^**^	0.03
Testcross parental trial-1	0.64^**^	0.05	−0.20	0.12	0.05	0.10	−0.43^**^	0.13	0.01	0.09	0.19	0.11	−0.37^**^
Testcross parental trial-2	0.81^**^	0.01	−0.23^*^	0.61^**^	0.13	0.41^**^	−0.53^**^	0.10	−0.20	0.53^**^	0.16	0.35^**^	−0.43^**^
Testcross parental trial-3	0.73^**^	−0.04	−0.13	−0.03	−0.29^*^	0.35^**^	−0.06	−0.01	−0.15	0.22	−0.25^*^	0.27^*^	−0.10
Testcross parental trial-4	0.81^**^	0.11	−0.14	−0.11	−0.16	0.27^*^	−0.17	0.17	−0.14	0.04	−0.03	0.09	−0.21
*Iniadi* accessions	0.74^**^	0.03	−0.12	0.34^**^	0.06	0.24^**^	−0.14	0.09	−0.16^*^	0.19^**^	0.05	0.14^*^	−0.11
Designated B-lines	0.75^**^	0.35^**^	0.00	0.26^*^	0.27^*^	0.50^**^	−0.01	0.44^**^	0.04	0.29^*^	0.31^**^	0.41^**^	0.00
Across trials	0.79^**^	0.14^**^	−0.06^*^	0.24^**^	0.23^**^	0.44^**^	−0.11^**^	0.08	−0.16^**^	0.22^**^	0.37^**^	0.53^**^	0.01

*,** *Significant at P < 0.05 and P < 0.01 probability, respectively*.

Fe and Zn showed mostly positive association with P, and the association was significant only in two trials for Fe (*r* = ≤ 0.40) and three trials for Zn (*r* = 0.36–0.47). Such significant association was observed in released cultivars and designated hybrid parents trials and not in other trials. The hypothesis is, such significant association exist due to homeostasis cross-talk between P, Zn, and Fe for better crop survival and fitness (47) as the designated parents and released cultivars consisted of well-adapted materials across regions [nitrogen (N), phosphorus (P) and potassium (K) (NPK) applications for better yield], while others are in pipeline testing and yet to be tested for wider adaption. Therefore, breeding for P improvement in hybrid parents and cultivar breeding is highly possible together with Fe and Zn in pearl millet. The association of Fe and Zn with Ca was mostly in the positive direction and significant in five trials with Fe (*r* = 0.26–0.61) and Zn (*r* = 0.21–0.53). While Mn showed a positive association with Fe and Zn in all the trials, a significant association was seen in 10 trials with Fe (*r* = 0.24–0.50) and eight trials with Zn (*r* = 0.14–0.52). This positive association of Fe and Zn with Mn, and to some extent with Ca, suggested the possibility of improving Fe and Zn along with these micronutrients. Positive or negative correlations of other minerals with Fe and Zn content were not always significant. For instance, Mg had a positive and significant association with Fe in two trials (*r* = 0.27–0.42) and with Zn in three trials (0.31–0.52), while the association was significant and negative in one trial (trial 11) with both Fe (*r* = −0.29) and Zn (*r* = −0.25). Na had a negative and significant association with Fe (*r* = −0.35 to −0.53) in four trials and with Zn (*r* = −0.37 to −0.51) in five trials. Fe and Zn had a significant negative association with K only in two trials (*r* = −0.16 to −0.40). On the contrary, Zn had a positive and significant association (*r* = 0.48) with K in one trial (trial 8). Very few germplasm-based studies in pearl millet revealed that Fe and Zn were significantly negatively correlated with P and no correlation was detected between Ca and Fe, Zn, and P (42, 48). The order and magnitude of the interrelationship of Fe and Zn with these grain minerals suggest that similar genetic and physiology/molecular mechanisms control Fe and Zn mobilization, uptake, distribution, and accumulation in pearl millet without much interference or adversely affecting the accumulation of other nutrients in the grain. These two micronutrients were weakly correlated with K and Na. Such weak and negative trait linkages can be broken using a directional selection in a larger segregating population (early generations). It can also be executed using genomic marker technology, identifying single-nucleotide polymorphism (SNP) markers associated with the target nutrient traits (so-called diagnostic markers) in early generation breeding pipelines. Interestingly, positive and significant associations of Fe and Zn with P, Ca, and Mn were observed in released cultivars (trial 6) and with P, Ca, Mg, and Mn across 14 trials that consisted of hybrids, inbreds, and germplasm. This implies that genetic improvement of productivity traits in advanced breeding lines, hybrid parents, and hybrids with micronutrients and macronutrients is highly feasible in pearl millet. Correlations indicate that Fe and Zn can be improved simultaneously with a few nutrients such as Ca and Mn. Capitalizing on the available latent genetic variation for grain nutrients and the absence of significant negative associations among these traits is a promising prospect for mainstreaming nutrition traits in pearl millet in the near future.

**Figure 3:**
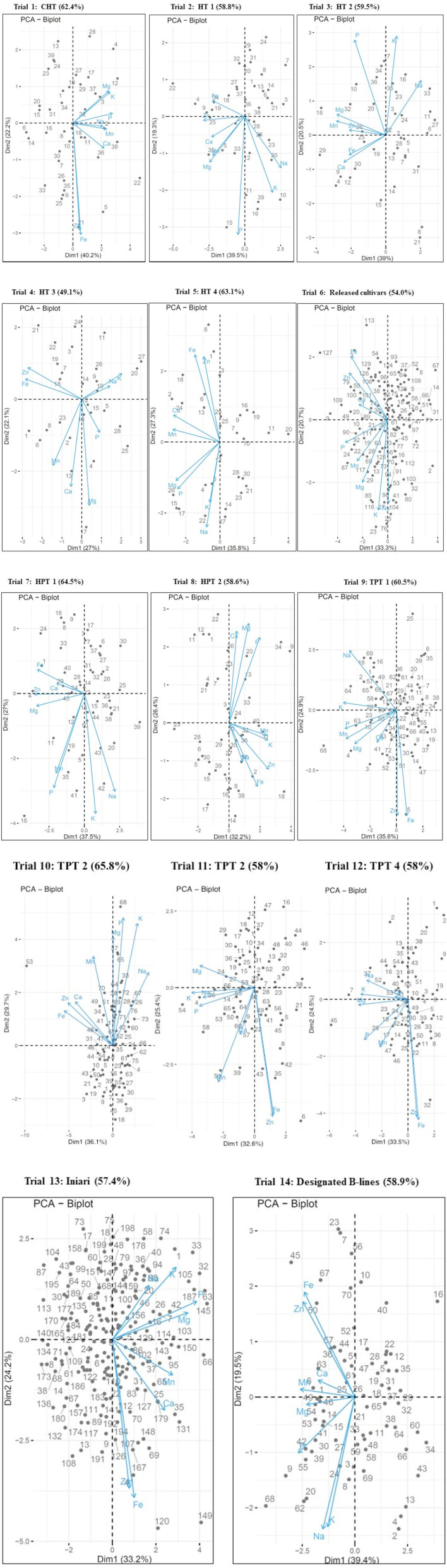
Genotype by trait (GT) biplot of 14 pearl millet trials for grain macronutrient and micronutrient densities.

Phenotypic correlation of pair traits among genotypes is largely dependent on previous breeding objectives for a given trait and likely to be misguided because of the effect of a different trait. Therefore, critical analysis and visualization are required to choose desired traits-based germplasm. This study demonstrated eight nutritional traits association across a different set of genotypes to guide multitrait selection strategy in pearl millet. The genotype (G) by trait (T) biplot is becoming a better tool for depicting correlation among traits across genotypes (35). The relationship among the nutritional traits studied in 14 trials using the GT biplot is shown in Figure 3. The GT biplot explained 49.1–63.1% of the total variation in hybrid trials, while it was 57.4–65.8% in parents and germplasm trials, suggesting substantial trait variations explained for each trial and confirming the significant variance in the analysis of variance. Longer vector lengths, mostly observed for Fe, Zn, P, K, and Na, indicate wide variations among test genotypes (in hybrids, parents, and germplasm), whereas the shorter vector length specifies minimal variation among genotypes for other traits in almost all the trials. In such cases, the use of three-dimensional plots may help to visualize the spread variations. It is important to note that the test materials originated from the regular breeding program chiefly targeted for improving productivity traits. The correlation coefficient among traits presented for each trial (Table 4) supports the results of the GT biplot with a similar direction. For instance, Fe and Zn were always positively associated in all the trials (Figure 3) except for one trial (HT-2), where the association was positive but weak. The positive relationship of both Fe and Zn with Ca and Mn is similar to the correlation matrix. Findings suggest that the breeding for Fe is likely to improve Zn in pearl millet, and interestingly the two nutrients traits can be genetically improved independently from other nutrients. GT biplot delineated the best genotypes as potential sources for one or more desired nutrient traits in pearl millet. A similar pattern of a positive association between Fe and Zn in the GT biplot was reported in pearl millet (49). The GT biplot serves as a quick breeder tool for the selection of nutri-dense entry for each of the nutrients as well as more than one nutrient can be selected for in potential parents (trait donor).

## Conclusion

This study revealed the potential genetic variation for eight pearl millet grain nutrients coupled with relatively high heritability and significant positive association especially with Fe and Zn content in diverse breeding materials (>900 entries). Released cultivars had low-to-moderate grain mineral variability in the diverse high-yielding backgrounds, suggesting the need for monitoring grain minerals in future cultivars. Parents and germplasm had higher nutrient content; the identified mineral-dense genotypes merit exploration for hybridization to improve grain mineral contents in next-generation breeding progenies and cultivar breeding. The findings of this study indicate that genetic selection for Fe will improve Zn as an associated trait in pearl millet. In addition, the Fe and Zn relationship with other macronutrients and micronutrients suggests increased prospects to achieve multiple nutritional gains in the ongoing HarvestPlus-supported pearl millet biofortification breeding. The results of this study warrant a further systematic multilocation testing to ascertain the magnitude of *G* × *E* interaction concerning micronutrient traits selection accuracy, stability, and inheritance to assist in devising appropriate breeding strategies.

## Data Availability Statement

The original contributions presented in the study are included in the article/Supplementary Files, further inquiries can be directed to the corresponding author.

## Author Contributions

MG planned the study and prepared the manuscript. AK and HS recorded the data and supported data analyses. KR and WP contributed the preparation of the manuscript. All the authors read and approved the final version of the manuscript.

## Funding

This study was supported by special project funding from the HarvestPlus Biofortification Challenge Program of the CGIAR. It was carried out as a part of the CGIAR Research Program (CRP) on Agriculture for Nutrition and Health (A4NH).

## Conflict of Interest

The authors declare that the research was conducted in the absence of any commercial or financial relationships that could be construed as a potential conflict of interest. The reviewer HO declared a shared affiliation, with no collaboration, with several of the authors MG, AK, KN, and HS.

## Publisher's Note

All claims expressed in this article are solely those of the authors and do not necessarily represent those of their affiliated organizations, or those of the publisher, the editors and the reviewers. Any product that may be evaluated in this article, or claim that may be made by its manufacturer, is not guaranteed or endorsed by the publisher.

## References

[1] Welch RM Graham RD. Breeding for micronutrients in staple food crops from a human nutrition perspective. J Exp Bot. (2004) 55:353–64. 10.1093/jxb/erh064 14739261

[2] Mayer JE Pfeiffer WH Beyer P. Biofortified crops to alleviate micronutrient malnutrition. Curr Opinion Plant Biol. (2008) 11:166–170. 10.1016/j.pbi.2008.01.007 18314378

[3] Narayan J Denny J Nirupama D. Malnutrition in India: status and government initiatives. J Public Health Policy. (2019) 40:126–41. 10.1057/s41271-018-0149-5. 10.1057/s41271-018-0149-5 30353132

[4] Nguyen PH Scott S Headey D Singh N Tran LM. The double burden of malnutrition in India: Trends and inequalities (2006–2016). PLoS ONE. (2021) 16:e0247856. 10.1371/journal.pone.0247856 33630964 PMC7906302

[5] Muthayya S Rah JH Sugimoto JD Roos FF Kraemer K. The Global Hidden Hunger Indices and maps: An advocacy tool for action. PLoS ONE. (2013) 8:e67860. 10.1371/journal.pone.0067860 23776712 PMC3680387

[6] Belay A Gashu D Joy EJM Lark R M Christopher C. Zinc deficiency is highly prevalent and spatially dependent over short distances in Ethiopia. Sci Rep. (2021) 11:6510. 10.1038/s41598-021-85977-x 33753836 PMC7985319

[7] Qaim M Stein AJ Meenakshi JV. Economics of biofortification. Agri Econ. (2007) 37:119–33. 10.1111/j.1574-0862.2007.00239.x

[8] Stein AJ Nestel P Meenakshi JV Qaim M Sachdev HPS. Plant breeding to control zinc deficiency in India: How cost-effective is biofortification? Public Health Nutr. (2007) 10:492–501. 10.1017/S1368980007223857 17411470

[9] Palanog AD Calayugan MIC Descalsota-Empleo GI Amparado A Inabangan-Asilo MA. Zinc and iron nutrition status in the Philippines population and local soils. Front Nutr. (2019) 6:81. 10.3389/fnut.2019.00081 31231657 PMC6568233

[10] Al-Fartusie FS Mohssan SN. Essential trace elements and their vital roles in human body. Indian J Adv Chem Sci. (2017) 5:127–136.15285149

[11] FAO/WHO. Preliminary report on recommended nutrient intakes. Joint FAO/WHO Expert Consultation on Human Vitamin and Mineral Requirements, FAO, Bangkok, Thailand. (2000). Geneva, Switzerland: Food and Agricultural Organization of the United Nations and World Health Organization. (accessed on Sept21–30, 1998).

[12] Knez M Stangoulis JCR. Calcium biofortification of crops-challenges and projected benefits. Front Plant Sci. (2021) 12:669053. 10.3389/fpls.2021.669053 34335646 PMC8323714

[13] Grusak M. A. Cakmak I. Methods to improve the crop-delivery of minerals to humans and livestock. In: Broadley MR., White PJ, editors. Plant Nutritional Genomics. (2005). Oxford: Blackwell Publishing. p. 265–86.

[14] Sands DC Morris CE Dratz EA Pilgeram AL. Elevating optimal human nutrition to a central goal of plant breeding and production of plant-based foods. Plant Sci. (2009) 177:377–89. 10.1016/j.plantsci.2009.07.011 20467463 PMC2866137

[15] Cakmak I Pfeiffer WH McClafferty B. Biofortification of durum wheat with zinc and iron. Cereal Chem. (2010) 87:10–20. 10.1094/CCHEM-87-1-001030812563

[16] Singh D Prasanna R. Potential of microbes in the biofortification of Zn and Fe in dietary food grains. Rev Agron Sustain Dev. (2020) 40:15. 10.1007/s13593-020-00619-2

[17] Grusak MA. Enhancing mineral content in plant food products. J Am College Nutr. (2002) 21:178S−83S. 10.1080/07315724.2002.10719263 12071302

[18] Virk PS Andersson MS Arcos J Govindaraj M Pfeiffer WH. Transition from targeted breeding to mainstreaming of biofortification traits in crop improvement programs. Front Plant Sci. (2021) 12:703990. 10.3389/fpls.2021.703990 34594348 PMC8477801

[19] Kumar S Hash CT Thirunavukkarasu N Singh G Rajaram V. Mapping quantitative trait loci controlling high iron and zinc content in self and open pollinated grains of pearl millet (*Pennisetum glaucum* (L) R Br). Front Plant Sci. (2016) 7:1636. 10.3389/fpls.2016.01636 27933068 PMC5120122

[20] AICRP on Pearl Millet. Report on the Project Coordinator Review 2019. 54th Annual Group Meeting, ICAR- Indian Agricultural Research Institute. New Delhi. (2019). p. 1–12.

[21] Basavaraj G Parthasarathy Rao P Bhagavatula S Ahmed W. Availability and utilization of pearl millet in India. J SAT Agri Res. (2010) 8:1–6.

[22] Parthasarathy RP Birthal PS Reddy BVS Rai KN Ramesh S. Diagnostics of sorghum and pearl millet grains-based nutrition in India International Sorghum and Millets. Newsletter. (2006) 46:93–6.

[23] Govindaraj M Rai KN Cherian B Pfeiffer WH Kanatti A. Breeding biofortified pearl millet varieties and hybrids to enhance millet markets for human nutrition. Agriculture. (2019) 9:106. 10.3390/agriculture9050106

[24] Guo S Chen Y Chen X Chen Y Yang L. Grain mineral accumulation changes in Chinese Maize Cultivars released in different decades and the responses to nitrogen fertilizer. Front Plant Sci. (2019) 10. 10.3389/fpls.2019.01662 31993065 PMC6971105

[25] Rosanoff A. Changing crop magnesium concentrations: impact on human health. Plant Soil. (2013) 368:139–53. 10.1007/s11104-012-1471-5

[26] Govindaraj M Rai KN Shanmugasundaram P. Intra-population genetic variance for grain iron and zinc contents and agronomic traits in pearl millet. Crop J. (2016) 04:48–54. 10.1016/j.cj.2015.11.002

[27] Pujar M Govindaraj M Gangaprasad S Kanatti A Shivade H. Genetic variation and diversity for grain iron, zinc, protein and agronomic traits in advanced breeding lines of pearl millet [*Pennisetum glaucum* (L.) R. Br.] for biofortification breeding. Genet Resour Crop Evol. (2020) 67:2009–22 10.1007/s10722-020-00956-x

[28] Govindaraj M Rai KN Shanmugasundaram P Dwivedi SL Sahrawat KL. Combining ability and heterosis for grain iron and zinc densities in pearl millet. Crop Sci. (2013) 53:507–17. 10.2135/cropsci2012.08.047725674488

[29] Wheal MS Fowles TO Palmer LT. A cost-effective acid digestion method using closed polypropylene tubes for inductively coupled plasma optical emission spectrometry (ICP-OES) analysis of plant essential elements. Anal Meth. (2011) 3:2854–63. 10.1039/c1ay05430a

[30] Yasmin Z Paltridge N Graham R Huynh B Stangoulis J. Measuring genotypic variation in wheat seed iron first requires stringent protocols to minimize soil iron contamination. Crop Sci. (2014) 54:255–64. 10.2135/cropsci2013.04.0214

[31] Gomez KA Gomez AA. Statistical procedures for agricultural research. 2nd ed (1984). John Wiley and Sons, New York.

[32] Hallauer AR Carena MJ Miranda Filho JB. Quantitative Genetics in Maize Breeding (third edition). (2010). New York, USA: Springer science, LLC. p. 10013.

[33] Al-Jibouri HA Miler PA Robinson HF. Genotypic and environmental variance and co-variance in an upland cotton cross of interspecific origin. Agron J. (1958) 50:633–6. 10.2134/agronj1958.00021962005000100020x

[34] Snedecor GW Cochran WG. Statistical Methods, Sixth edition. (1967). The Iowa State University Press, Ames, IA.

[35] Yan W. GGEbiplot: a windows application for graphical analysis of multi-environment trial data and types of two-way data. Agron J. (2001) 93:1111–8. 10.2134/agronj2001.9351111x

[36] Velu G Rai KN Muralidharan V Kulkarni VN Longvah T. Prospects of breeding biofortified pearl millet with high grain iron and zinc content. Plant Breeding. (2007) 126:182–5. 10.1111/j.1439-0523.2007.01322.x33173120

[37] Velu G Rai KN Sahrawat KL. Variability for grain iron and zinc content in a diverse range of pearl millet populations. Crop Improve. (2008) 35:186–91.

[38] Gupta SK Velu G Rai KN Sumalini K. Association of grain iron and zinc content with grain yield and other traits in pearl millet (*Pennisetum glaucum* (L.) R. Br.). Crop Improve. (2009) 36:4–7.

[39] Govindaraj M Selvi B Sudhir KI. Genetic diversity studies in indigenous pearl millet [*Pennisetum glauccum (*L) R *Br*] accessions based on biometrical and nutritional quality traits. Indian J Plant Genetic Res. (2011) 24:186–93.

[40] Govindaraj M Rai KN Shanmugasundaram P Rao AS. Efficiency of single plant selection for grain iron and zinc density in pearl millet. Eur J Plant Sci Biotechnol. (2012) 6:114–7.

[41] Velu G. Genetic Variability, Stability and Inheritance of Grain Iron and Zinc Content in Pearl Millet (Pennisetum glaucum (L.) R. Br.). Ph.D., Thesis, Tamil Nadu Agricultural University, Coimbatore, India.

[42] Arulselvi S Mohanasundaram K Selvi B. Genetic analysis of grain quality characters and grain yield in pearl millet *(Pennisetum glaucum (L.) R. Br.)*. Crop Res. (2009) 37:161–167.

[43] Velu G Rai KN Muralidharan V Longvah T Crossa J. Gene effects and heterosis for grain iron and zinc density in pearl millet (*Pennisetum glaucum (L) R Br*). Euphytica. (2011) 180:251–9. 10.1007/s10681-011-0387-0

[44] Rai KN Govindaraj M Rao AS. Genetic enhancement of grain iron and zinc content in pearl millet. Qual Assur Safety Crops Foods. (2012) 4:119–25. 10.1111/j.1757-837X.2012.00135.x34589092

[45] Kanatti A Rai KN Radhika K Govindaraj M Sahrawat KL. Grain iron and zinc density in pearl millet: combining ability, heterosis and association with grain yield and grain size. Springerplus. (2014) 3:763. 10.1186/2193-1801-3-763 25674488 PMC4320223

[46] Kanatti A Rai KN Radhika K Govindaraj M Rao AS. Genetic architecture of open-pollinated varieties of pearl millet for grain iron and zinc densities. Indian J Genetics Plant Breed. (2016) 76:299–303. 10.5958/0975-6906.2016.00045.6

[47] Xie X Hu W Fan X Chen H Tang M. Interactions Between Phosphorus, Zinc, and Iron Homeostasis in Nonmycorrhizal and Mycorrhizal Plants. Front Plant Sci. (2019) 10:1172. 10.3389/fpls.2019.01172 31616454 PMC6775243

[48] Govindaraj M Selvi B Rajarathinam S. Correlation studies for grain yield components and nutritional quality traits in pearl millet (*Pennisetum glaucum* (L.) R. Br.) germplasm. World J Agri Sci. (2009) 5: 686–689.

[49] Sanjana Reddy P Satyavathi CT Khandelwal V Patil HT Gupta PC Sharma LD. Performance and stability of pearl millet varieties for grain yield and micronutrients in arid and semi-arid regions of India. Front Plant Sci. (2021) 12:1–16. 10.3389/fpls.2021.670201 34135925 PMC8202413

