# Supplementary material for: Association of Grain Iron and Zinc Content With Other Nutrients in Pearl Millet Germplasm, Breeding Lines, and Hybrids
Source: Front Nutr. 2022 Feb 2;8:746625. doi: 10.3389/fnut.2021.746625 (PMC8847779; doi:10.3389/fnut.2021.746625)
Supplement: Supplementary file 1 [file Table_1.pdf]

Supplementary table 1. Mean and variability for grain macronutrient density in pearl millet breeding trials at ICRISAT, Hyderabad, India.

Trial name	P (mg 100 g^{-1})		K (mg 100 g^{-1})		Ca (mg 100 g^{-1})		Mg (mg 100 g^{-1})	
	Mean	Range	Mean	Range	Mean	Range	Mean	Range
Hybrid trials								
Commercial hybrid trial	374	325 - 435	500	405 - 600	10	6.5 - 16.0	135	113 - 168
Hybrid trial-1	357	330 - 395	492	425 - 575	10	6.8 - 15.1	120	108 - 132
Hybrid trial-2	370	325 - 415	492	450 - 535	11	8.7 - 16.3	124	109 - 149
Hybrid trial-3	371	345 - 400	473	430 - 555	10	7.0 - 17.8	125	113 - 142
Hybrid trial-4	379	335 - 410	484	415 - 550	10	4.2 - 16.5	133	112 - 152
Released cultivar trial	330	275 - 380	415	350 - 530	12	7.8 - 17.8	124	102 - 157
Breeding/Parental lines								
Hybrid parental trial-1	361	313 - 450	500	397 - 663	14	7.5 - 28.0	118	94 - 151
Hybrid parental trial-2	384	330 - 467	518	363 - 633	15	7.9 - 26.0	130	98 - 165
Testcross parental trial-1	366	310 - 445	503	405 - 660	14	5.1 - 29.0	126	96 - 172
Testcross parental trial-2	363	310 - 445	487	355 - 670	15	8.3 - 40.0	130	95 - 171
Testcross parental trial-3	366	295 - 420	485	355 - 695	13	7.7 - 24.0	129	104 - 163
Testcross parental trial-4	377	315 - 470	513	390 - 725	14	6.5 - 28.5	136	107 - 177
Iniadi accessions	387	330 - 475	430	340 - 550	13	7.5 - 35.0	144	117 - 189
Designated B-lines	378	310 - 495	552	410 - 675	12	6.8 - 20.5	140	109 - 174
Across trials	369	275 - 495	489	340 - 725	12	4.2 - 40.0	130	94 - 189

Supplementary table 2. Mean and variability for grain micronutrient density in pearl millet breeding trials at ICRISAT, Hyderabad, India.

.

Trial name	Fe (mg kg^{-1})		Zn (mg kg^{-1})		Mn (mg kg^{-1})		Na (mg kg^{-1})	
	Mean	Range	Mean	Range	Mean	Range	Mean	Range
Hybrid trials								
Commercial hybrid trial	42	31 - 53	35	26 - 45	12	10 - 16	16	7 - 29
Hybrid trial-1	54	39 - 68	37	31 - 49	12	9 - 15	13	6 - 20
Hybrid trial-2	54	43 - 68	38	32 - 49	12	10 - 17	14	10 - 24
Hybrid trial-3	48	36 - 61	38	29 - 47	12	10 - 16	11	7 - 19
Hybrid trial-4	47	38 - 60	37	30 - 44	12	9 - 15	11	7 - 19
Released cultivar trial	49	34 - 82	42	29 - 57	14	11 - 19	15	7- 29
Breeding/Parental lines								
Hybrid parental trial-1	62	35 - 93	42	26 - 62	11	8 - 20	12	3 - 30
Hybrid parental trial-2	53	30 - 83	39	27 - 64	11	7 - 18	13	3 - 31
Testcross parental trial-1	55	32 - 98	41	24 - 60	12	8 - 20	11	4 - 24
Testcross parental trial-2	56	27 - 145	42	26 - 95	13	8 - 23	11	3 - 22
Testcross parental trial-3	51	30 - 100	40	25 - 64	12	8 - 18	11	3 - 26
Testcross parental trial-4	56	29 - 94	42	22 - 70	13	8 - 19	12	5 - 23
Iniadi accessions	72	44 - 119	62	40 - 96	16	10 - 21	18	5- 41
Designated B-lines	50	24 - 80	37	23 - 49	13	8 - 17	14	8– 25
Across trials	53	24-145	41	22-96	13	7-23	13	3-41
